# Supplementary material for: Strong structuring arising from weak cooperative O-H···π and C-H···O hydrogen bonding in benzene-methanol solution
Source: Nat Commun. 2023 Sep 22;14:5900. doi: 10.1038/s41467-023-41451-y (PMC10516861; doi:10.1038/s41467-023-41451-y)
Supplement: Supplementary file 1 — Supplementary Information [file 41467_2023_41451_MOESM1_ESM.pdf]

Supplementary Information: Strong structuring arising from weak cooperative O-H···π and C-H···O hydrogen bonding in benzene-methanol solution

Camilla Di Mino,[a] Andrew G. Seel,[b,*] Adam J. Clancy,[c] Thomas F. Headen,[b] Támas Földes,[a] Edina Rosta,[a] Andrea Sella,[c,*] Neal T. Skipper,[a,*]

(a) Department of Physics and Astronomy, University College London, London WC1E 6BT (UK)

(b) ISIS Neutron and Muon Source, Science and Technology Facilities Council, Rutherford Appleton Laboratory, Didcot, OX11 0QX (UK)

(c) Department of Chemistry, University College London, 20 Gordon Street, London WC1H 0AJ (UK)

Email: n.skipper@ucl.ac.uk; a.sella@ucl.ac.uk; andrew.seel@stfc.ac.uk.

Supplementary Note 1. Neutron Diffraction Theory

Neutron diffraction combined with isotopic substitution is a powerful tool for the study of liquid structures. Specifically, the difference in coherent neutron scattering lengths (and therefore respective scattering cross sections) between hydrogen (b_h = -3.74 fm) and deuterium (b_d = 6.67 fm) can be exploited to obtain multiple distinct scattering profiles for a given liquid system. [1]

In a neutron diffraction experiment of an isotropic system such as a liquid, one can obtain the isotopically unique structure factor; [2]

$$F(Q) = \sum_{\alpha,\beta \geq \alpha} \left(2 - \delta_{\alpha\,\beta}\right) b_\alpha\, b_\beta\, c_\alpha\, c_\beta\, \left(S_{\alpha\beta}(Q)\ -\ 1\,\right) \tag{1}$$

where c_α and c_β, b_α and b_β are respectively the fractional concentrations of the atomic species α and β and the coherent scattering lengths, $Q = 4\pi\frac{\sin\theta}{\lambda}$ is the magnitude of the neutron scattering vector, and $S_{\alpha\beta}(Q)$ the Faber-Ziman partial structure factor for any two types of atoms. We can then rewrite supplementary equation 1 as follows:

$$F(Q) = \sum_{\alpha,\beta\geq\alpha} w_{\alpha\beta}\left(S_{\alpha\beta}(Q) - 1\right) \tag{2}$$

where $w_{\alpha\,\beta}$ are the terms in the weights matrix, calculated for each sample in Supplementary Table 1.

The Fourier Transformation of the Partial Structure Factor in supplementary equation 1 and 2 is:

$$S_{\alpha\beta}(Q) - 1 = \frac{4\pi\rho_0}{Q}\int_0^\infty r\left[g_{\alpha\beta}(r) - 1\right]\sin(Qr)\,dr \tag{3}$$

where ρ_0 is the atomic density of the system and $g_{\alpha\beta}(r)$ are the partial pair distribution functions representing the relative probability density of finding an atom of species β at a

distance *r* from an atom of species α as the origin of the reference system. In liquid systems, where there is no long-range order, the $g_{\alpha\beta}(r)$ in supplementary equation 3 therefore have an asymptote of 1 at large-*r* and give important site-specific structural information of the system.[3] The total radial distribution function *G(r)* is the weighted sum of the partial distribution functions $g_{\alpha\beta}(r)$ and is related to the structure factor *F(Q)* by the Fourier transform:

$$G(r) = \frac{1}{(2\pi)^3\rho_0}\int_0^\infty 4\pi Q^2 F(Q)\frac{\sin(Qr)}{Qr}dQ = \sum_{\alpha,\beta\geq\alpha}(2-\delta_{\alpha\beta})b_\alpha b_\beta c_\alpha c_\beta\left(g_{\alpha\beta}(r)-1\right). \quad (4)$$

In order to quantify the average coordination number, $N_{\alpha\beta}(r_0)$, of sites of type *β* in proximity to a site of type *α* up to a maximum distance r_0, one can integrate the partial radial distribution function $g_{\alpha\beta}(r)$ over the separation distance, *r*

$$N_{\alpha\beta}(r_0) = 4\pi\int_0^{r_0} g_{\alpha\beta}(r)\rho_\beta r^2 dr. \quad (5)$$

Note that the integration limit r_0 is traditionally the first minimum of the partial radial distribution function.

Supplementary Table 1. Neutron Weights. *Neutron weighting coefficients calculated from supplementary equation 2 for the six isotopically distinct data sets.*

	C_6D_6 in CH_3OD	C_6D_6 in CD_3OH	C_6H_6 in CH_3OD	C_6D_6 / C_6H_6 in CD_3OH	C_6D_6 / C_6H_6 in CD_3OD	C_6D_6 in CD_3OD
C_M-H	0.01009	-0.00566	0.01009	-0.00566	0.01099	0.01099
C_M-O	0.00878	0.00878	0.00878	0.00878	0.00878	0.00878
C_M-C_M	0.01006	0.01006	0.01006	0.01006	0.01006	0.01006
C_M-H_M	-0.01697	0.03027	0.03027	0.03027	0.03027	0.03027
H_M-H_M	0.02860	0.09107	0.02860	0.09107	0.02860	0.02860
H_M-H	-0.01702	-0.01702	0.03057	-0.01702	0.03057	0.03057
H_M-O	-0.01481	0.02641	0.02641	0.02641	0.02641	0.02641
H-H	0.01012	0.03182	0.01012	0.03182	0.01012	0.01012
H-O	0.00880	-0.04936	0.00880	-0.04936	0.00880	0.00880
O-O	0.00766	0.00766	0.00766	0.00766	0.00766	0.00766
C_1-C_1	0.00100	0.00100	0.00100	0.00100	0.00100	0.00100
C_1-H_1	0.00101	0.00101	-0.00056	0.00022	0.00022	0.00101
H_1-H_1	0.00101	0.00101	0.00032	0.00003	0.00003	0.00101
C_1-C_M	0.00032	0.00032	0.00032	0.00032	0.00032	0.00032
C_1-O	0.00028	0.00028	0.00028	0.00028	0.00028	0.00028
C_1-H	0.00319	-0.00179	0.00319	-0.00179	0.00319	0.00319
C_1-H_M	-0.00536	0.00956	0.00956	0.00956	0.00956	0.00956
H_1-C_M	0.003186	0.003186	-0.00179	0.00134	0.00134	0.003186
H_1-O	0.00278	0.00278	-0.00156	0.00117	0.00117	0.00278
H_1-H	0.00319	-0.00179	-0.00179	0.00079	0.00140	0.00320
H_1-H_M	-0.00538	0.00959	-0.00538	0.00421	0.00421	0.00959

Supplementary Note 2. The Empirical Potential Structure Refinement (EPSR) Method [4]

The EPSR method consists of Monte Carlo simulation which takes initial seed potentials for modelling pairwise interactions, and subsequently refines these through the incorporation of an empirical potential until a satisfactory agreement between the calculated structure factors and experimental neutron scattering data is reached. In this manner, a three-dimensional structural model of the system can be obtained which is consistent with the experimental data. The seed potentials used are based on a Lennard-Jones 12-6 function plus a Coulomb term where appropriate:

$$U_{inter} = U_{\alpha\beta}(r_{ij}) = 4\epsilon_{\alpha\beta}\left[\left(\frac{\sigma_{\alpha\beta}}{r_{ij}}\right)^{12} - \left(\frac{\sigma_{\alpha\beta}}{r_{ij}}\right)^{6}\right] + \frac{q_\alpha q_\beta}{4\pi\epsilon_0 r_{ij}} \tag{6}$$

where the well depth parameter $\epsilon_{\alpha\beta}$ and the range parameter $\sigma_{\alpha\beta}$ are given for a multicomponent system by the Lorentz-Berthelot mixing rules

$$\epsilon_{\alpha\beta} = \sqrt{\epsilon_\alpha \epsilon_\beta}\,; \sigma_{\alpha\beta} = \frac{1}{2}(\sigma_\alpha + \sigma_\beta).\ ^{5} \tag{7}$$

The input potentials used for benzene are OPLS-AA while the methanol molecule is modelled via a three-site model developed by Jorgensen and co-workers and are summarised in Supplementary Table 2.[6,7] During the EPSR procedures, the benzene molecule is planar and rigid, whilst the methanol is allowed to rotate freely. The cubic EPSR box of sides 41.95 Å contains a total of 1000 molecules in the ratio 1:19 benzene:methanol (50 benzenes, 950 methanols) with atomic number density of 0.09 atoms $Å^3$. This model of the system reproduces the experimental composition and density and is sufficiently large to capture possible solute-solute, solute-solvent and solvent-solvent intermolecular interactions.

The labels assigned to atomic sites on the benzene and methanol molecules are shown in Supplementary Figure 1. The simulation was brought to equilibrium with an initial 2,000 iterations, and then the Empirical Potential refinement switched on. Once a good agreement between data and model was achieved, a trajectory file was accumulated for approximately 100,000 configurations. All the structural information of the system contained in $g_{\alpha\beta}(r)s$, ARDFs, SDFs were then extracted and visualised using dlputils and Aten packages.[8,9]

Supplementary Table 2. Lennard-Jones Seed Parameters and Charges for Methanol and Benzene. *OPLS atom-atom potentials and charges for benzene. OPLS united atom potentials and charges for methanol (J2 model).*[6,7]

	**Atom Type**	σ /Å	ϵ /kJ mol^{-1}	***q*** /e
**Methanol**	C_M	3.775	0.86609	0.297
	H_M	-	-	-
	O	3.070	0.71128	-0.700
	H	-	-	0.435
**Benzene**	C_1	3.55	0.29288	-0.115
	H_1	2.42	0.12552	0.115

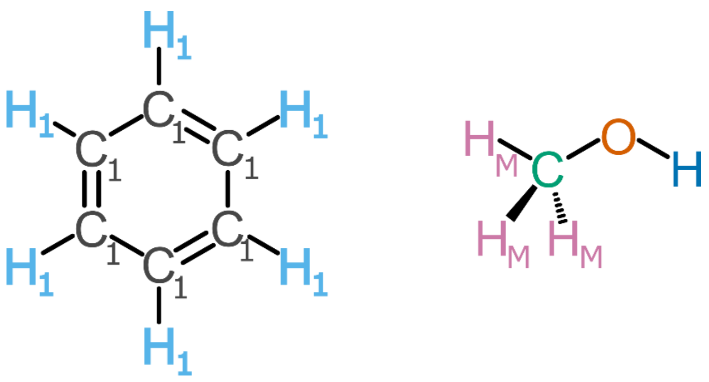

Supplementary Figure 1. Benzene and Methanol Atoms Labelling. *EPSR labelling for the different atomic species for benzene (left) and methanol (right).*

Reference measurements on pure methanol were obtained during the same experiment and the complete data set may be found at https://data.isis.stfc.ac.uk/doi/study/103207982. An

EPSR simulation with 500 methanol molecules was performed to obtain the partial $g_{\alpha\beta}(r)s$ of Figure 3, Supplementary Figure 2 and 3. A structural analysis of pure methanol using the EPSR method has already been published.[10]

Supplementary Note 3. Total and Partial Radial Distribution Functions

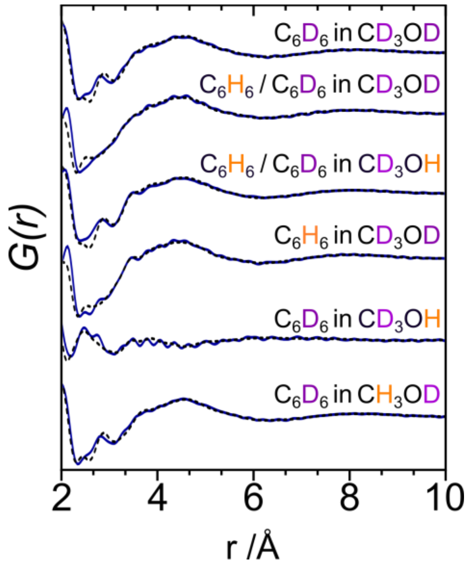

Supplementary Figure 2. Total Radial Distribution Function G(r) in the 2 – 10 Å Range. *Total radial distribution functions of Figure 1 main text, but plotted now from 2 Å to 10 Å. The features in the G(r)s at short distances are dominated by the well-reproduced intramolecular contributions, while those at longer distances arise from intermolecular interactions and are broader due to the intrinsic nature of liquid systems. Experimental (solid) and EPSR modelled (dashed).*

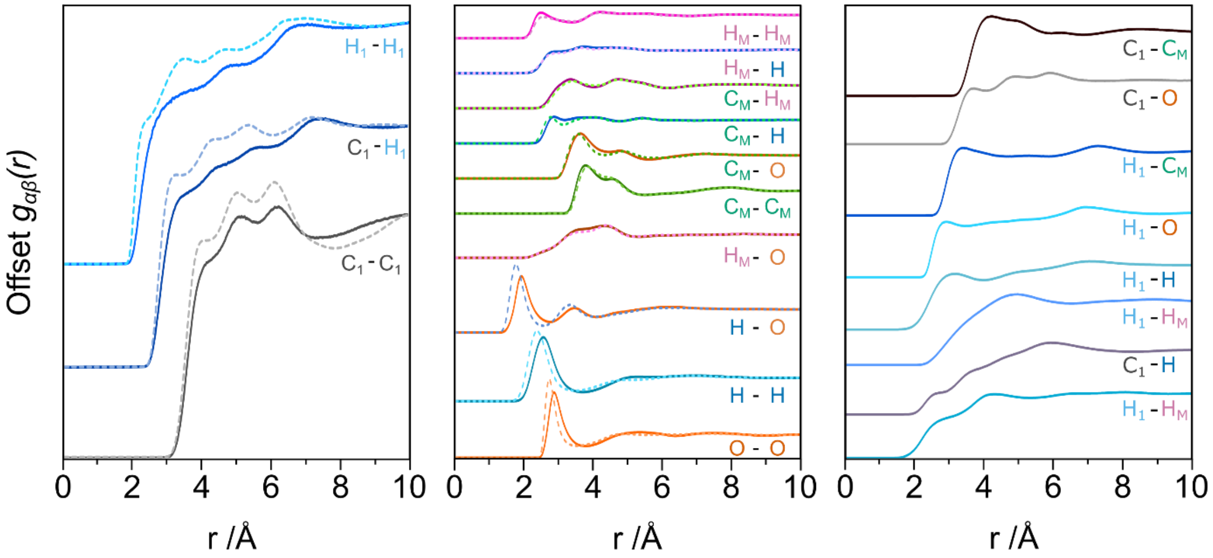

Supplementary Figure 3. Additional Partial Distribution Functions for Benzene-Benzene, Methanol-Methanol and Benzene-Methanol. *Offset intermolecular partial radial distribution functions, $g_{\alpha\beta}(r)$, for benzene-benzene and methanol-methanol in the pure liquid (dotted lines) and in the 1:19 benzene-methanol mixture (solid lines). On mixing, changes to the underlying site-site functions of benzene and methanol are relatively subtle.*

Supplementary Note 4. Angular Radial Distribution Functions and Spatial Distribution Functions

To evaluate the relative orientation of the methanol molecule around the benzene, we calculated the Angular Radial Distribution Functions (ARDFs). The ARDFs are the partial distribution functions calculated as a function of the relative orientation of the two species considered;

$$g(r,\theta) = \frac{\Delta n(r,\theta)}{\frac{2}{3}\pi((r+\Delta r)^3 - r^3)\sin(\theta)\Delta\theta\rho} \quad (7)$$

where $\Delta n(r,\theta)$ is the number of molecules at distance $r + \Delta r$ and orientation $\theta + \Delta\theta$, ρ is the atomic number density. In our analysis we put benzene in the centre of the frame of reference, where *x* and *y* are the in-plane vectors and the *z* vector is parallel to the axial vector of the benzene. The methanol molecule main axes are O-H and C-O, as shown in Supplementary Figure 4.

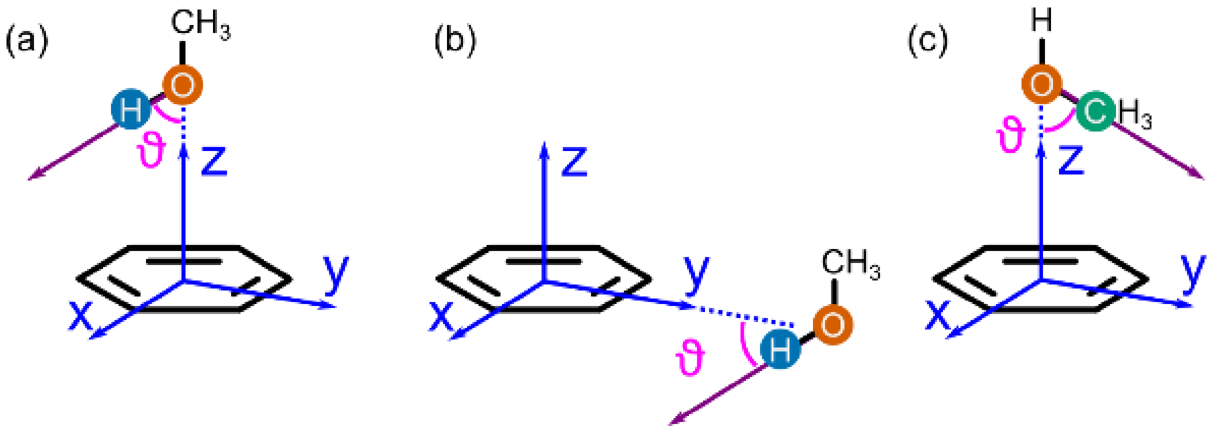

Supplementary Figure 4. Benzene and Methanol Axes Definition. *Coordinate system used for the Angular Radial Distribution Functions (ARDFs) of Figure 3. Relative orientation of a) the benzene principal* C_6 *axis (defined as z) and the O-H vector on the methanol; b) the benzene x axis and the O-H vector on the methanol; c) the benzene principal* C_6 *axis (defined as z) and the C-O vector on the methanol.*

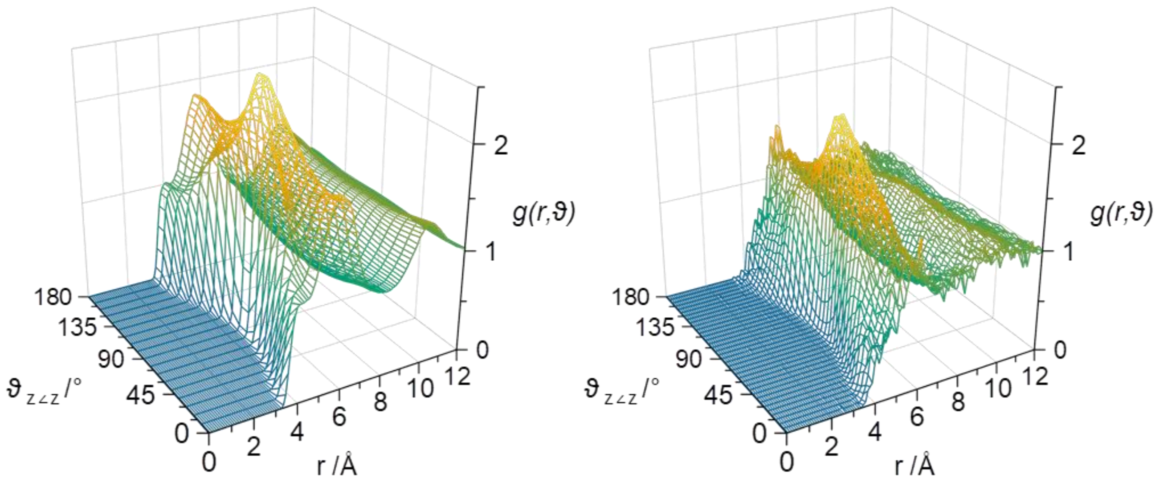

Supplementary Figure 5. Angular Radial Distribution Function of Pure Benzene and Benzene in Methanol. *Angular Radial Distribution Functions (ARDFs) relative orientation of the benzene principal C_6 axis (defined as z) and the z axis of a different benzene molecule in bulk benzene (left) and in 1:19 solution with methanol. Please note the different $\boldsymbol{g(r,\theta)}$ scales.*

Supplementary Figure 5 presents the ARDFs of the relative orientation of the C_6 axis of two distinct benzene molecules in bulk benzene and in the 1:19 benzene-methanol mixture. The two ARDFs are similar, as they present a first weak peak at 0˚ and 180˚ due to parallel-displaced motifs, and, at further distances, they show a sharp well-defined peak at 90˚ indicating the perpendicular “Y” motif. The relative intensities of the peaks indicate that the benzene-benzene interaction is weaker in the methanol mixture than in the pure liquid, and this observation is consistent with the information extracted from the partial distribution functions of Figure 2 and the respective benzene-benzene coordination number of 1.2 (Table 1).

The Spatial Density Functions (SDFs) are a three-dimensional map of the density of neighbouring molecules around a central molecule as a function of angular distance, *r,* and angular position ϑ. The SDFs therefore represent regions of space around a central molecule that are most likely to be occupied by a molecule of the same or another species in a given distance. The 3D grid associated with the SDFs is plotted by filling in the voxels from most probable downwards and this process is stopped when cumulative probability reaches

a set percentage for the volume over the SDFs were calculated. In the case of the SDFs of Figure 5 a_2, b_2 and c_2 main text the probability is 30%, 5% and 10% respectively. All the SDFs were produced defining the axes as shown in Supplementary Figure 4.

To calculate the probability distribution for the number of benzene CoM - methanol H contacts out-of-plane, we used the dlputils routine to count molecule-by-molecule over the total number of frames (79630) within 3.5 Å distance. [8]

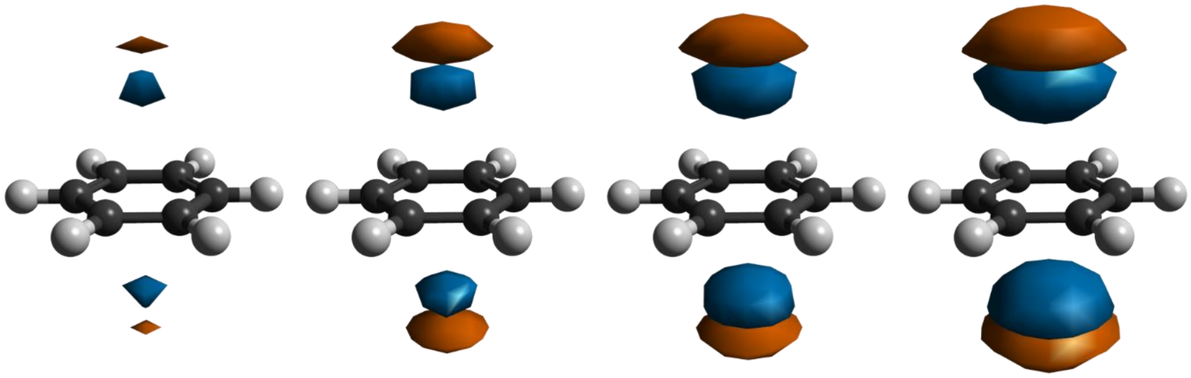

Supplementary Figure 6. Out-of-Plane Spatial Density Functions at Different Visualization Percentages. *Spatial Density Functions (SDFs) showing the 10%, 30%, 50% and 70% most likely positions of methanol hydroxyl H (dark blue) and methanol O (dark orange) up to 3.1 Å and 3.7 Å from the benzene CoM. Note the definition and the directionality of these density functions.*

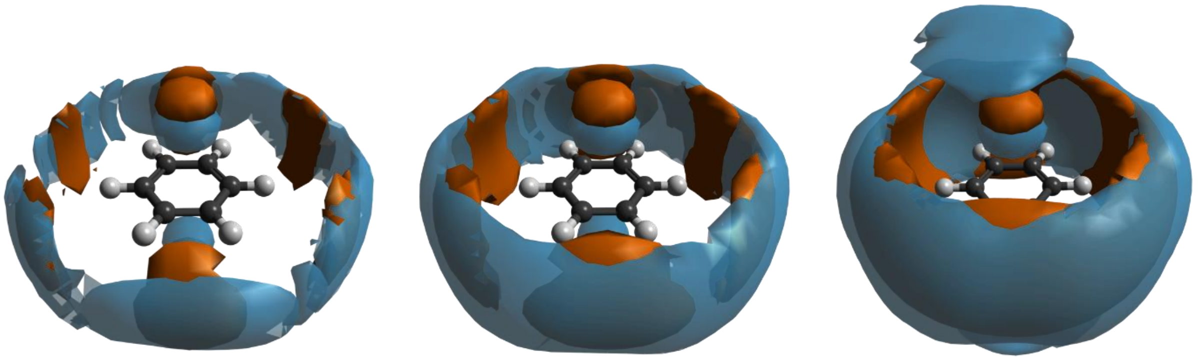

Supplementary Figure 7. Full First Solvation Spatial Density Functions at Different Visualization Percentages. *Spatial Density Functions (SDFs) showing the 5%, 10% and 30% most likely positions of methanol hydroxyl H (dark blue) and methanol O (dark orange) up to 6.8 from the benzene CoM.*

Supplementary Note 5. Molecular Dynamics (MD) and Monte Carlo (MC) Simulations without empirical potential refinement

The classical Molecular Dynamics (MD) simulations were performed via the NAMD2.13 package using the CHARMM36m force field (CGenFF).[11,12] The molecular structures were initially optimized via the Gaussian09 software at the B3LYP-D3/6-311G (d,p) level of theory in vacuum. Restricted Partial atomic charges (RESP) for all atoms for the benzene and methanol molecules were calculated at the HF/6-31G* level of theory with the Merz-Singh-Kollman method.[13,14] The input files for the MD simulation were generated with CHARMM-GUI.[15,16] After 10000 steps of minimisation, the system was equilibrated for 1ns of simulation time in a constant volume at 298.15K temperature. The subsequent production run was carried out for 110ns at 298.15K and 1atm. Electrostatic interactions were treated using the Particle Mesh Ewald summation with a cut-off distance of 12 Å.

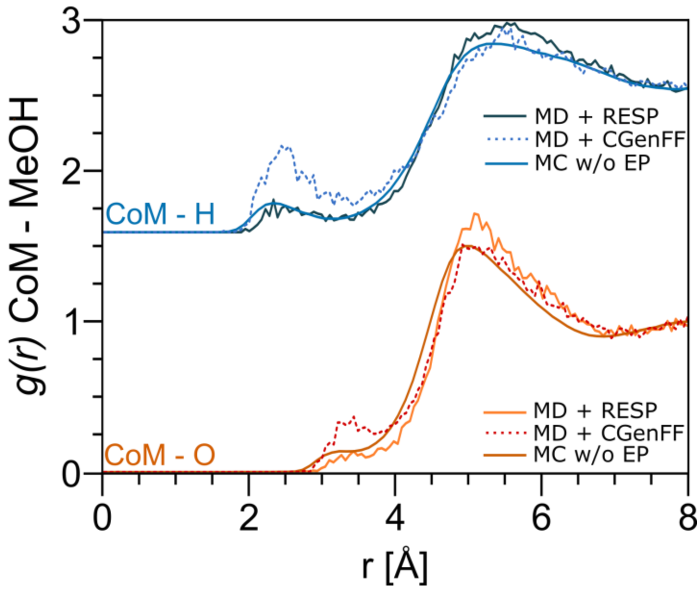

Supplementary Figure 8. Comparison between Partial Distribution Functions from Unrefined Classical Models. *Partial radial distribution functions benzene Centre-of-Mass (CoM) – methanol H (blue) and O (orange) for the MD and the MC without empirical potential (w/o EP) simulations.*

Supplementary Table 3. Comparison between Partial Distribution Functions Peak Positions from Unrefined Classical Models. *Partial radial distribution functions peak positions extracted from the MD and MC w/o EP simulations.*

	g(r) **CoM – H 1st Peak Position /Å**	***g(r)*** **CoM – H 2nd Peak Position / Å**	***g(r)*** **CoM – O 1st Peak Position / Å**	***g(r)*** **CoM – O 2nd Peak Position / Å**
**EPSR refined**	2.30	4.90	3.25	4.90
**MC OPLS-AA w/o EP**	2.38	5.35	3.39	5.10
**MD CHARMM + RESP**	2.57	5.50	3.45	5.10
**MD CHARMM + CGenFF**	2.55	5.58	3.47	5.13

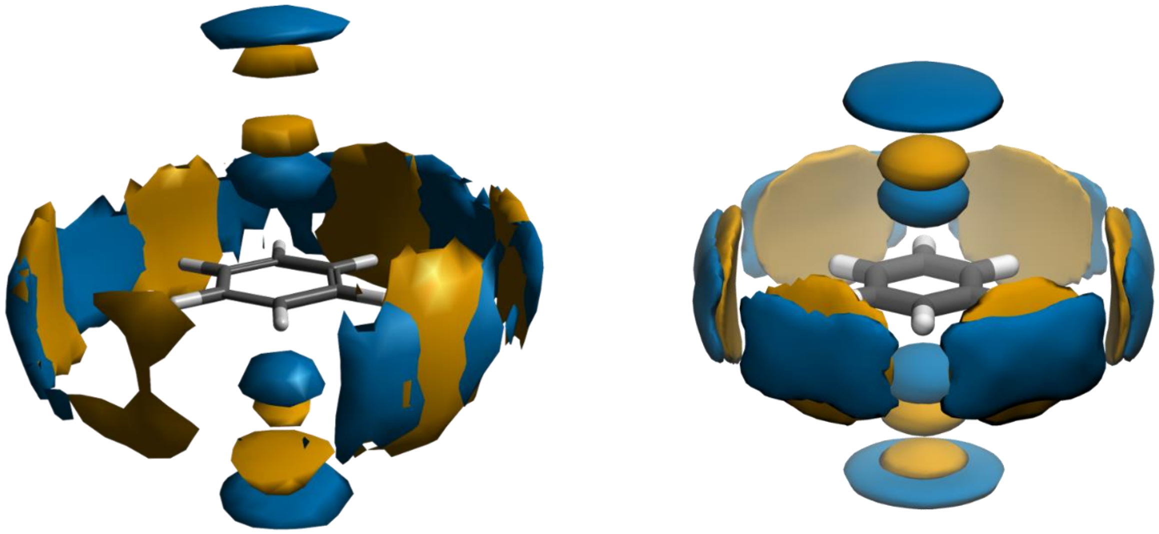

Supplementary Figure 9. Full First Solvation Spatial Density Functions from unrefined classical models. *Spatial Distribution Function (SDFs) showing methanol H (blue) and O (orange) up to 7 Å distance from the benzene CoM. Both MD (RESP charges) and MC (w/o EP) simulations show distinct bifurcated C-H···O interaction, however the hydrogen is located at further distances, underestimating the role of cooperation for these important structural motifs.*[17]

Supplementary References

1. Sears, V. F. Neutron scattering lengths and cross sections. *Neutron News* **3**, 26–37 (1992).

2. Fischer, H. E., Barnes, A. C. & Salmon, P. S. Neutron and x-ray diffraction studies of liquids and glasses. *Rep. Prog. Phys.* **69**, 233 (2005).

3. Terban, M. W. & Billinge, S. J. L. Structural analysis of molecular materials using the pair distribution function. *Chem. Rev.* **122**, 1208–1272 (2022).

4. Soper, A. K. Empirical potential Monte Carlo simulation of fluid structure. *Chem. Phys.* **202**, 295–306 (1996).

5. Lorentz, H. A. Ueber die Anwendung des Satzes vom Virial in der kinetischen Theorie der Gase. *Ann Phys* **248**, 127–136 (1881).

6. Jorgensen, W. L., Maxwell, D. S. & Tirado-Rives, J. Development and Testing of the OPLS All-Atom Force Field on Conformational Energetics and Properties of Organic Liquids. *J. Am. Chem. Soc.* **118**, 11225–11236 (1996).

7. Jorgensen, W. L. Optimized intermolecular potential functions for liquid alcohols. *J. Phys. Chem.* **90**, 1276–1284 (1986).

8. Youngs, T. G. A. dlputils (GitHub, 2022).

9. Youngs, T. G. A. Aten-An application for the creation, editing, and visualization of coordinates for glasses, liquids, crystals, and molecules. *J. Comp. Chem.* **31**, 639–648 (2010).

10. Yamaguchi, T., Hidaka, K. & Soper, A. K. The structure of liquid methanol revisited: a neutron diffraction experiment at -80 C and +25 C. *Mol. Phys.* **96**, 1159–1168 (1999).

11. Phillips, J. C. *et al.* Scalable molecular dynamics on CPU and GPU architectures with NAMD. *J Chem Phys* **153**, 44130 (2020).

12. Huang, J. *et al.* CHARMM36m: an improved force field for folded and intrinsically disordered proteins. *Nat. Methods* **14**, 71–73 (2017).

13. Singh, U. C. & Kollman, P. A. An approach to computing electrostatic charges for molecules. *J. Comput. Chem.* **5**, 129–145 (1984).

14. Besler, B. H., Merz Jr, K. M. & Kollman, P. A. Atomic charges derived from semiempirical methods. *J. Comput. Chem.* **11**, 431–439 (1990).

15. Jo, S., Kim, T., Iyer, V. G. & Im, W. CHARMM-GUI: a web-based graphical user interface for CHARMM. *J. Comput. Chem.* **29**, 1859–1865 (2008).

16. Lee, J. *et al.* CHARMM-GUI input generator for NAMD, GROMACS, AMBER, OpenMM, and CHARMM/OpenMM simulations using the CHARMM36 additive force field. *J. Chem. Theory Comput.* **12**, 405–413 (2016).

17. Mahadevi, A. S. & Sastry, G. N. Cooperativity in Noncovalent Interactions. *Chem. Rev.* **116**, 2775–2825 (2016).
